# Descriptive analysis of 1,048 presentations in the first five years of a zero-suicide framework in a child and youth mental health service in Australia

**DOI:** 10.3389/fpsyt.2024.1437016

**Published:** 2024-10-04

**Authors:** Grace Branjerdporn, Laura K. McCosker, Derek Jackson, Kerri M. Gillespie, Sarah McDowell, Sandeep Chand, Hitesh Joshi, Anthony R. Pisani, Nicolas J. C. Stapelberg, Matthew Welch, Kathryn Turner, Sabine Woerwag-Mehta

**Affiliations:** ^1^ Mental Health and Specialist Services, Gold Coast Hospital and Health Service, Southport, QLD, Australia; ^2^ Faculty of Health Sciences and Medicine, Bond University, Robina, QLD, Australia; ^3^ Departments of Psychiatry and Pediatrics, Center for the Study and Prevention of Suicide, University of Rochester, Rochester, NY, United States; ^4^ Mental Health and Specialist Services, Metro North Hospital and Health Service, Brisbane, QLD, Australia

**Keywords:** zero suicide framework, suicide prevention, youth, adolescent, child, mental health

## Abstract

**Introduction:**

Suicide in children is a significant and unacceptable global phenomenon. This paper provides a descriptive overview of the children presenting in the first five years (2016-2021) of the implementation of the Zero Suicide Framework (ZSF) and Suicide Prevention Pathway (SPP) at a Child and Youth Mental Health Service in Queensland, Australia.

**Methods:**

Basic demographic variables (sex, age, socioeconomic status), and changes in presentations over time, are presented for 1,048 children. Completeness of selected SPP components relating to care planning and universal interventions are examined as an indicator of fidelity to the SPP model of care. The paper then focuses on the cohort of children who received care through the SPP in 2020, describing their demographic characteristics and baseline clinical scores.

**Result:**

There was an increase in admissions each year and children presented with a diverse range of clinical needs. The SPP greatly increased the provision of first line interventions for patients.

**Discussion:**

A standardized approach to suicide prevention improves consistency in management. These findings may inform the use of the ZSF/SPP in child mental health settings globally.

## 1 Introduction

### 1.1 Suicide in children

A ‘suicide’ is defined as a death caused by a person who injures themselves with the intention to die (1). Each year, globally, more than 700,000 people of all ages die by suicide (2). In Australia, suicide is the leading cause of death among young people aged 15-19 years (3). For each suicide, there are approximately 20 more people who attempt suicide (4, 5). Suicide attempts and suicidal ideation among children (people aged ≤17 years) in particular are significant and growing problems in Australia, with young people having the highest rates of hospitalizations for self-harm (5). A large national study found that around 7.5% of young people aged 12 to 17 had seriously considered suicide, and 2.4% had attempted suicide in the past 12 months (6). Prevention is vitally important.

### 1.2 The Gold Coast Mental Health and Specialist Service

The Gold Coast Mental Health and Specialist Service (GCMHSS) in Queensland, Australia sees >5,400 suicidal and self-harm presentations (including suicide attempts, suicidal ideation, and non-suicidal self-injury) each year via its two hospital emergency departments (EDs) (7). Suicidal and self-harm presentations increased from 1,446 or 1.3% of all ED presentations in 2009 to 5,380 or 3.1% of all ED presentations in 2018. In the same period, young people aged 15-24 years accounted for 37.4% of suicidal and self-harm presentations by females and 28.1% of presentations by males (7). Children aged ≤17 years of age at risk of suicide are referred to the Child and Youth Mental Health Service (CYMHS) within GCMHSS. The CYMHS provides 24-hour multidisciplinary care to children with severe and complex problems, including (but not limited to) suicidality (i.e. suicide ideation and attempts) (8, 9).

### 1.3 The Suicide Prevention Pathway

In 2016, the GCMHSS CYMHS began utilizing the Zero Suicide Framework (ZSF) approach (10) to guide clinical practice. The ZSF was originally developed by the National Action Alliance for Suicide Prevention (10) and adapted at GCMHSS for the Australian setting. It focuses on suicide prevention interventions delivered through whole systems of care where access, quality, and safety are continuously reviewed and improved (11, 12).

The ZSF is implemented in clinical practice through the Suicide Prevention Pathway (SPP). Children and young people who attempt suicide are eligible for the SPP, which involves five sequential components: (a) screening to identify suicide risk; (b) chronological assessment of suicide events (CASE); (c) risk formulation to better understand risk; (d) universal interventions aimed at enhancing safety (including lethal means counselling and safety planning), and; (e) a structured follow-up and transition of care (13). Risk formulation refers to the Pisani method of prevention-oriented risk formulation (14). This formulation is anchored in the clinical context and considers a person’s mental state compared to their baseline (risk state), compared to a specified community or clinical subpopulation (risk status), and incorporates potential exacerbating or resilience factors. At the GCMHSS CYMHS, each component has been carefully adapted to meet the particular needs of children; we have described this process in detail elsewhere (15). The focus is on a culture of no blame, collaboration, and recovery (16). This differs from traditional interventions which often focus on treating the medical consequences of a child’s suicide attempt and then discharge without sufficient follow up (16).

This paper provides a descriptive overview of the children presenting in the first five years (December 2016-2021) of the implementation of the ZSF and SPP at the GCMHSS CYMHS. Our primary goal is to obtain an overview of the SPP model of care and characteristics of children entering the service. We will focus on three measurable components of the model to describe fidelity to the SPP model of care: risk formulation, safety planning and lethal means counselling. The Case approach is a set of interview techniques specifically designed to put a person with suicidality at ease to talk about their current and past suicide event(s). These interview techniques or “soft skills” are not captured in a case file. It would require live observation or recording to measure whether these techniques are applied as intended. Live observation or video recording of all children in crisis with suicidality is not feasible and in addition, our lived experience work force raised concerns that this would be stressful for a child and their parents and counterproductive to the engagement process. The elicited content with the CASE approach varies from child to child and hence cannot be standardized and assessed for completeness. The child’s account is reflected and integrated in the risk formulation, safety planning and lethal means counselling. These three standardized components have clearly defined components, which can be measured for completeness. For these children, basic demographic variables (sex, age) are presented and changes over time analyzed. The paper then focuses on the cohort of children who received care through the SPP in 2020, describing their demographic characteristics and baseline clinical scores. The aim of the paper is to inform the use of the ZSF/SPP in child mental health settings elsewhere in Australia and globally.

## 2 Materials and methods

This study was exempt from ethical review as it involves routine data collection undertaken for clinical quality assurance (Gold Coast Health Human Research Ethics Committee (HREC) Reference LNR/2018/QGC/47473, 11 October 2018; and EX/2021/QGC/79427, 8 October 2021). Part 1 of the study provides a summary of the children receiving care through the SPP between 2016 and 2021. It includes all children (≤17 years of age) who: (a) presented to CYMHS between December 01, 2016, and December 31, 2021, and (b) received care through the SPP. It looks at each child’s first presentation to the CYMHS only. Data focused on mental health characteristics associated with first suicide attempt presentations and treatment episode requiring the SPP. Clinical observation suggests that young people’s mental health characteristics worsen or remain unresolved with suicide attempt representations and hence multiple attempters are a clinically distinct subgroup, which may affect the generalizability of the findings (17). Data about each child’s: (a) demographic variables, (b) SPP journey, including the completeness of selected SPP components, and (c) total clinical scores, were extracted manually from Queensland Health’s Consumer Integrated Mental Health and Addiction (CIMHA) application.

Part 2 of the study focuses on the cohort of children receiving care through the SPP in 2020. It includes all children (≤17 years of age) who: (a) presented to CYMHS between January 01 and December 31, 2020, and (b) received care through this pathway, and (c) had results recorded for one or more of the following mental health screening tools: the *Health of the Nations Outcome Scales for Children and Adolescents* (HoNOSCA), and/or the *Child Global Assessment Scale* (CGAS), and/or the *Strength and Difficulties Questionnaires* (self-rated and parent-rated versions, SDQ-SR and SDQ-P). The HoNOSCA is a clinician-rated, 15-item assessment which summarizes a patients’ functioning within four subscales: behavior, impairment, symptoms and social (18). Similarly, the CGAS is a clinician-rated assessment which scores the patient from 1 (very poor global functioning) to 100 (excellent global functioning) with anchoring descriptors for each score decile (19). The SDQ is completed by both child and their parent/carer. It assesses 25 psychological attributes across five domains: emotion, conduct, hyperactivity, peer problems, and prosocial behavior (20).

Data about each child’s: (a) demographic variables, and (b) mental health screening tool scores, were extracted from CIMHA. Cut-off points for clinically significant scores on screening tools were obtained from published literature. Based on their suburb of residence at first presentation, each child was matched with a Socio-Economic Indexes for Areas (SEIFA) percentile, based on Australian Bureau of Statistics data (21). Higher SEIFA scores represent higher socio-economic advantage. Each child’s primary diagnosis was determined based on the International Statistical Classification of Diseases and Related Health Problems, Tenth Revision, Australian Modification (ICD-10-AM). Only the final diagnosis for each child (where relevant) is reported. A diagnosis can change over the course of admission and therefore the final diagnosis is considered to be the most reliable.

Data were analyzed in SPSS version 28.0.1.1 (22). Data were analyzed descriptively, using simple counts and proportions. If relevant, means, standard deviations, minimums, and maximums were calculated.

## 3 Results

### 3.1 Summary of SPP presentations, 2016-2021

Between December 2016 and December 2021, 1,048 children received care through the SPP at GCMHSS. Of these, 173 (16.5%) re-presented in the timeframe. The following analysis is based on each young person’s first presentation (i.e., the first time they received care through the SPP).

**Figure 1:**
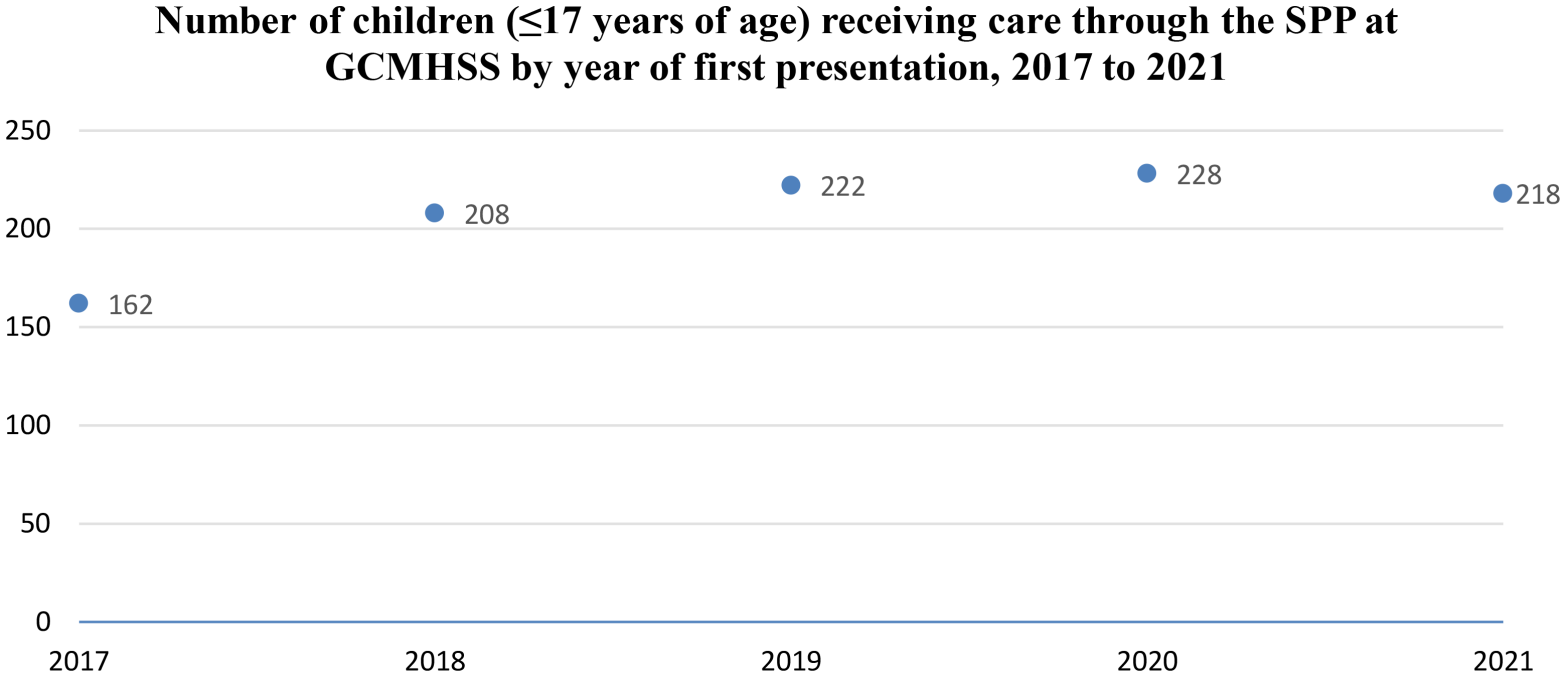
Number of children (≤17 years of age) receiving care through the SPP at GCMHSS by year of first presentation, December 2016 to December 2021.

From 2017 to 2020, the number of children receiving care through the SPP increased year-on-year (**Figure 1**). Year 2016 was not included in the figure as only one month of data was collected. There was a slight decrease in 2021, the second full year of the COVID-19 pandemic:

A total of 1,017 (97%) young people had their gender recorded at their first presentation. Of these, most identified as female (N=694, 68.2%) (**Table 1**). Approximately one-third (n=317, 31.2%) identified as male, and a small minority identified as ‘other’ (N=6, 0.6%). All the children had age recorded when they first received care through the SPP. More than two-thirds (n=77, 67.4%) were aged ≥15 years. The largest age-group was the 16-year-olds (n=243, 23.2%), followed by the 15- and 17-year-olds (n=232, 22.1% respectively):

**Table 1:** Gender and age of the children (≤17 years of age) receiving care through the SPP at GCMHSS by year of first presentation, December 2016 to December 2021.

	2016* n (%)	2017 n (%)	2018 n (%)	2019 n (%)	2020 n (%)	2021 n (%)
Male	4 (40)	44 (27.2)	79 (38)	75 (33.8)	74 (32.5)	41 (21.9)
Female	6 (60)	118 (72.8)	128 (61.5)	143 (64.4)	153 (67.1)	146 (78.1)
Unknown	0	0 (0.0)	1 (0.5)	4 (1.8)	1 (0.4)	0
TOTAL N (%)	10 (100)	162 (100)	208 (100)	222 (100)	228 (100)	187 (100)
6 years	0 (0.0)	0 (0.0)	1 (0.5)	0	1 (0.4)	0
7 years	0 (0.0)	0 (0.0)	0	0	0	0
8 years	0 (0.0)	1 (0.6)	0	1 (0.5)	0	0
9 years	0 (0.0)	0 (0.0)	2 (1)	1 (0.5)	1 (0.4)	1 (0.5)
10 years	0 (0.0)	0 (0.0)	1 (0.5)	3 (1.4)	1 (0.4)	0
11 years	0 (0.0)	2 (1.2)	3 (1.4)	5 (2.3)	4 (1.8)	2 (1.1)
12 years	0 (0.0)	6 (3.7)	14 (6.7)	13 (5.9)	10 (4.4)	15 (8)
13 years	1 (10)	14 (8.6)	21 (10.1)	22 (9.9)	16 (7)	29 (15.5)
14 years	2 (20)	19 (11.7)	26 (12.5)	28 (12.6)	42 (18.4)	33 (17.6)
15 years	1 (10)	38 (23.5)	43 (20.7)	45 (20.3)	50 (21.9)	55 (19.4)
16 years	3 (30)	44 (27.2)	41 (19.7)	52 (23.4)	58 (25.4)	45 (24.1)
17 years	3 (30)	38 (23.5)	56 (26.9)	52 (23.4)	45 (19.7)	38 (20.3)
**TOTAL N (%)**	**10 (100)**	**162 (100)**	**208 (100)**	**222 (100)**	**228 (100)**	**218 (100)**

*December only.

### 3.2 Fidelity to the SPP model

**Table 2** shows completeness of three selected SPP components and subcomponents, for children with scores recorded. The 2015 data are for the year *prior* to the implementation of the SPP; it is from a cross-sectional service evaluation conducted in March/April 2015. The 2019/2020 data are for the third and fourth full years *after* the implementation of the SPP (i.e., after the two-year implementation period), and therefore more reflective of an established service model. For all of the selected components, there was an increase in completeness over time:

**Table 2:** Provision of first line suicide prevention interventions for children (≤17 years of age), 2015 (pre-implementation) and 2019-2020 (post-implementation) and the increase in use of a risk formulation in 2019 and 2020.

SPP Component	2015 Total n (n, % with outcome complete)	2019 Total n (n, % with outcome complete)	2020 Total n (n, % with outcome complete)
Suicide risk formulation	132 (N/A)	222 (177, 79.7)	216 (184, 85.2)
Safety plan	132 (15, 11.4)	222 (193, 86.9)	219 (181, 82.6)
Lethal means counselling	132 (15, 11.4)	222 (168, 75.7)	219 (167, 76.3)

There were five children who were known to CYMHS who died from suicide between 2016 and 2021. Of these children, three had received care through the SPP.

### 3.3 Focus on the 2020 cohort

Data form 2020 were further investigated. This year was chosen as the SPP model had passed its initial implementation and refinement period and would better reflect the outcomes of the established model of care. There were a total of 243 first or subsequent presentations of children receiving care through the SPP at CYMHS in 2020. Of these, 146 children (60.1%) had results recorded for one or more of the HoNOSCA, CGAS, or SDQ at their first or subsequent presentation and are included in this section. See **Table 3** for demographic characteristics of the study participants.

**Table 3:** Demographic characteristics of the study participants in 2020.

		*n* (%)	Mean (SD)	Min.	Max
Age (years)		146 (100)	15.1 (1.6)	11	17
Sex		146 (100)			
Female		102 (69.9)			
Male		44 (30.1)			
Socioeconomic status^+^		146 (100)	58.0 (20.5)	2	97
External referrals to the service		146 (100)	3.9 (3.8)	1	21
Country/continent of birth		146 (100)			
Australia		123 (84.3)			
New Zealand		11 (7.5)			
Asia		5 (3.4)			
United Kingdom		5 (3.4)			
North America		1 (0.7)			
Africa		1 (0.7)			
Indigenous status		146 (100)			
Neither Aboriginal nor Torres Strait Islander		139 (95.2)			
Aboriginal and/or Torres Strait Islander		6 (4.1)			
Not stated/unknown		1 (0.7)			
Diagnosis^++^		146 (100)			
F10-F19	Psychoactive substance use disorder	4 (2.7)			
F20-F29	Schizotypal/delusional disorder	1 (0.7)			
F30-F39	Affective-related disorder	25 (17.1)			
F40-F49	Anxiety-related disorder	39 (26.7)			
F50-F59	Behavioral-related disorder	2 (1.4)			
F60-F69	Personality-related disorders	13 (8.9)			
F70-F79	Mental retardation	1 (0.7)			
F80-F89	Developmental-related disorder	5 (3.4)			
F90-F98	Emotional-related disorder	25 (17.1)			
F99	Unspecified mental disorder	8 (5.5)			
R45.81	Homicidal/suicidal ideation	13 (8.9)			
	Other disorders	7 (4.8)			
	No diagnosis recorded	3 (2.1)			

^+^Suburb was used to rank the patient’s social-economic status percentile in Australia, according to the Socio-Economic Indexes for Areas (SEIFA),

^++^Diagnosis according to International Classification of Diseases, Tenth Revision, Australian Modification (ICD-10-AM).

#### 3.3.1 HoNOSCA

Rate of completion of the questions on the HoNOSCA tool was over 95% (see **Table 4**). The questions with the greatest proportion of clinically significant scores were Q9 Emotional symptoms (97.2%, n=141), Q3 Self-injury (68.3%, n=66), Q12 Family problems (66.0%, n=95), and Q10 Peer relationships (54.2%, n=78). The questions with the greatest proportion of non-clinically significant scores were Q6 Physical illness or disability (89.6, n=129), Q7 Hallucinations and delusions (86.9%, n=126), Q15 Lack of information about treatment (85.3%, n=112), and Q11 Self-care and independence problems (83.4%, n=121).

**Table 4:** Health of the Nations Outcome Scales for Children and Adolescents (HoNOSCA) scores for study participants in 2020.

	*n* (%)	Mean (SD)	Min.	Max.	Clinically Significant *N* (%)
Emotional symptoms	145 (99.3)	2.8 (0.8)	0	4	141 (97.2)
Self-injury	145 (97.3)	2.0 (1.2)	0	4	99 (68.3)
Family problem	144 (98.6)	2.0 (1.2)	0	4	95 (66)
Peer relationship problem	144 (98.6)	1.6 (1.3)	0	4	78 (54.2)
Overactive, attention difficulty	144 (99.3)	1.2 (1.2)	0	4	56 (38.9)
Poor school attendance	139 (95.2)	1.0 (1.3)	0	4	48 (34.5)
Disruptive, aggressive problem	145 (98.6)	0.9 (1.2)	0	4	39 (26.9)
Psychosomatic problem	144 (98.6)	0.7 (1.1)	0	4	37 (25.7)
Alcohol, drug misuse	142 (97.3)	0.7 (1.2)	0	4	32 (22.5)
Lack of knowledge about difficulties	142 (97.3)	0.6 (1)	0	4	31 (21.8)
Scholastic or language skills problem	142 (98.6)	0.6 (1)	0	3	25 (17.6)
Self-care, independence problem	145 (99.3)	0.5 (0.9)	0	4	24 (16.6)
Lack of information about treatment	143 (97.6)	0.5 (0.8)	0	4	21 (14.7)
Hallucinations, delusions	145 (99.3)	0.4 (0.8)	0	4	19 (13.1)
Physical illness, disability problem	144 (98.6)	0.3 (0.8)	0	3	15 (10.4)

A ‘clinically significant’ score is a score of ≥2.

#### 3.3.2 CGAS

All 146 children (100.0%) completed the CGAS. The mean score was 58.8 (SD=12.4, range=31-90), falling into the range of ‘Some noticeable problems’, but not considered clinically-significant. Forty-three (29.5%) of the children had a clinically significant score on the CGAS.

#### 3.3.3 SDQ-SR and SDQ-P

One hundred and forty-three (97.9%) of the children completed the self-rated SDQ questionnaire (see **Table 5**). Most children (83.2%, n=119) recorded a total self-rated score that was clinically significant. The questions with the greatest proportion of clinically significant scores were Impact (94.1%, n=128) and Emotional (74.8%, n=107). The questions with the greatest proportion of non-clinically significant scores were Prosocial (72.2%, n=104) and Conduct (53.1%, n=76).

**Table 5:** Strength and Difficulties Questionnaire (SDQ) scores for study participants in 2020.

	*n* (%)	Mean (SD)	Min.	Max.	Clinically Significant *N* (%)
Youth					
Impact	143 (97.9)	5.1 (3.0)	1	10	128 (94.1)
Emotional	143 (97.9)	6.9 (2.1)	1	10	107 (74.8)
Hyperactivity	143 (97.9)	6.7 (2.4)	0	10	98 (68.5)
Peer	143 (97.9)	3.9 (2.1)	0	9	78 (54.5)
Conduct	143 (97.9)	3.5 (2.4)	0	9	67 (46.9)
Prosocial	143 (97.9)	6.9 (2.1)	1	10	39 (27.3)
*Total score*	143 (97.9)	21 (5.8)	5	36	119 (83.2)
Impact – Distress	137 (93.8)	1.3 (0.8)	0	2	
Impact – Home	137 (93.8)	1 (0.8)	0	2	
Impact – Friend	136 (93.2)	0.9 (0.9)	0	2	
Impact – Class	137 (93.8)	1.3 (0.8)	0	2	
Impact – Leisure	137 (93.8)	0.9 (0.8)	0	2	
Primary caregiver					
Impact	100 (68.5)	5.6 (3.4)	1	10	90 (93.8)
Emotional	99 (67.8)	6.7 (2.4)	0	10	88 (88.9)
Peer	99 (67.8)	3.8 (2.3)	1	10	67 (68.4)
Conduct	99 (67.8)	3.5 (2.5)	0	9	59 (59.6)
Hyperactivity	99 (67.8)	5.9 (2.5)	0	10	57 (57.6)
Prosocial	99 (67.8)	6.5 (2.5)	1	10	33 (33.3)
*Total score*	99 (67.8)	19.9 (6.8)	2	33	82 (82.8)

Up to 100 (68.5%) of the children had a parent/carer who completed the parent SDQ questionnaire. Most parents/carers (82.8%, n=82) recorded a total score that was clinically significant. The questions with the greatest proportion of clinically significant scores were Impact (93.5%, n=90) and Emotional (88.9%, n=88). The questions with the greatest proportion of non-clinically significant scores were Prosocial (66.7%, n=55) and Hyperactivity (42.4%, n=42). Impact subscale scores recorded by the children were further analyzed. There were particularly high impacts in relation to the young person’s experience of distress, and their engagement in class (i.e. education or training).

## 4 Discussion

This paper has provided a descriptive overview of the children (aged ≤17 years) presenting in the first five years (2016-2021) of the implementation of the ZSF and the SPP at a (CYMHS on the Gold Coast in Queensland, Australia. It has shown that the children presenting are diverse, in terms of both their demographic characteristics and their clinical needs. However, the paper has also demonstrated that a standardized care approach to suicide prevention improves consistency in the delivery of first line interventions. This is an important area for future research.

The number of children receiving care through the SPP at the GCMHSS CYMHS increased year-on-year from 2016. This is consistent with the upwards trend seen in hospitalizations of young people due to intentional self-harm in Australia (5). However, it also likely reflects an improvement in CYMHS clinicians’ awareness of and ability to elicit suicidal presentations and subsequent use of the SPP over time.

There was a slight decrease in the number of children receiving care through the SPP in 2021, the second (but, in terms of infection rate, worst to date) year of the COVID-19 pandemic in Australia. This is interesting, considering rates of mental illness and demand for mental health services in the general Australian population increased significantly throughout 2021 as a result of the pandemic (23). One possibility is that the reduction in the number of children receiving care through the SPP in 2021 was due to an increase in the burden the pandemic created for clinicians, rather than a decrease in suicidal presentations. During the first six months of the pandemic in Australia (March-August 2020), in comparison to the same six month period in 2019, there were significantly more suicidal and self-harm presentations by people aged <18 years to EDs in the Gold Coast Hospital and Health Service (16.9% vs 15.1%, *p*=0.041) (24), of which the GCMHSS is a part. Increased burden may have resulted in a lack of time available for clinicians to identify non-overt suicidality and complete the administrative tasks that enable a child to receive care through the SPP. Further, to meet high demand and cover staff losses due to illness, staff from other areas of the service were deployed to CYMHS, and may not have been trained in all aspects of the SPP. These issues might also explain why only 60.1% of the 2020 cohort had a HoNOSCA, CGAS, or SDQ score at their first presentation.

Most children receiving care through the SPP in the study period identified as female. In the general Australian population the suicide rate is higher in males (25), however rates of intentional self-harm are higher in females (26), and this reflects our findings. Interestingly, among the children aged ≤11 years in our study there were a greater number of males. This might be indicative of an inverse relationship between gender and suicidality in younger children, though it may also be an anomaly due to our small sample size. Indeed, other recent Australian research about suicidality in children suggests in the younger age-groups there may be a greater number of females (27). The relationship between gender and suicidality in younger children is an important topic for future research.

The average child receiving care through the SPP was in the 58^th^ percentile of advantage/disadvantage measured by SEIFA, and suburbs across the Gold Coast region average in the 57^th^ percentile (25). This falls into the ‘less disadvantaged’ category – interesting, considering that in Australia, intentional self-harm tends to be higher in areas of greater socio-economic disadvantage (28). Approximately 4.8% of the children receiving care through the SPP during the study period identified as Aboriginal and/or Torres Strait Islander; high, considering just 3.7% of young people (aged ≤24 years) on the Gold Coast identify as the same (29). This reflects the fact that in the general Australian population, the suicide rate is higher in people who identify as Aboriginal and/or Torres Strait Islander, as per Australia’s 2021 Census (30). Rates of intentional self-harm in First Nations Australians are also seen to be around three times higher than in non-Indigenous Australians (31). Finally, among the children receiving care through the SPP in the study period, the most common final diagnosis was an anxiety-related disorder; this is again consistent with trends in the broader Australian population (32).

Completeness of the three selected SPP components analyzed in this study – suicide risk formulation, safety planning, and lethal means counselling – increased over time. This reflects improvement in fidelity to the SPP, as clinicians become more accustomed to using it. Fidelity to the model is vital if we are to draw conclusions about its effectiveness. As can be seen, a standardized approach to suicide prevention has the potential to significantly increase the provision of first line interventions such as safety planning and access to lethal means counselling and fidelity to the model of care.

It is important to acknowledge that the approach and tools used in the SPP do not always completely prevent suicides, although this is desired, and we recorded three suicides among children receiving care through the SPP. The SPP does not involve a suicide-specific screening tool; indeed, a systematic review has found that there are currently no predictive tools for suicide that are clinically useful (33). The ZSF recommends tools such as the Colombia Suicide Severity Rating Scale (S-SSRS); however, this scale has not been rigorously validated in children (34). The SPP does not involve suicide risk stratification, as this has been found not to be predictive of a suicide attempt (35). The SPP includes follow-up sessions, though these are not discussed in this overview of the model as they include the same elements are reiterative and similar in format to the initial session. Follow-up sessions will continue to collect information that may not have been accessible in a crisis situation, reflect on past strategies, incorporate new strategies as required, and expand upon safety planning. Once the young person is stabilized, follow-up sessions will be used to make plans with the family and young person to transition to appropriate future care.

It is interesting that children receiving care through the SPP during the study period had assessment tool scores (HoNOSCA, CGAS, SDQ) that were both clinically significant for mental illness, and not clinically significant. In particular, the CGAS scores show that most of the children’s global functioning is reported not to be compromised to a clinically significant level. The SDQ and HONOSCA identified high levels of clinically significant scores across a range of domains. High proportions of children had psychosocial difficulties that greatly interfered with home life, friendships, classroom learning, and leisure activities (i.e., SDQ impact score). Additionally, many of the children presented with feelings of worry and nervousness (i.e., SDQ emotional difficulties and HoNOSCA emotional symptoms). It is plausible that these children have dysfunctional patterns of emotional regulation, with suicide attempts as a way of coping with their distressing emotions (36).

A high proportion of children had distractibility and difficulties with completing tasks (i.e., SDQ hyperactivity). This is congruent with other research showing associations between hyperactivity (specifically, attention deficit hyperactivity disorder) and suicidality (37). Most of the children had difficulties with being kind and considerate of others (i.e., SDQ prosocial scale), as well as making friends and being bullied (i.e., SDQ peer problems scale). This confirms similar findings revealing links between bullying, irritability, and defiance and suicidal behaviors (38, 39). Clinicians identified 66% of children had family difficulties. This finding aligns with other research showing that, in children, attachment difficulties, marital dysfunction, and suicidality are associated (40, 41).

Around 9% children receiving care through the SPP during the study period received a final diagnosis of R45.81 homicidal/suicidal ideation. As this is the final diagnosis at the end of the service episode, these children will have had ongoing suicidal ideation despite receiving care under the SPP. Analysis of the characteristics of children with ongoing suicidal ideation is important for improving ongoing care, and for developing targeted preventive strategies. Children who present with suicide attempts receive specialized care related to suicide prevention, as described in this study. However, opportunities to review these services should be utilized to explore the patterns of mental health issues experienced by children and young people who present with suicidal ideation, suicide attempts and self-harm in order to identify and address the numerous factors that are associated with, and may precede, suicidality. Additional strategies to identify these factors early should be considered in this model of care to ensure that vulnerabilities that contribute to the risk of suicidality (i.e., subsequent suicide attempts) are proactively managed.

### 4.1 Limitations

There are a number of limitations of the SPP at GCMHSS CYMHS. Children are initially screened in EDs. If suicide risk is not identified in EDs, children may not be referred to CYMHS and may have no opportunity to receive care through the SPP. The implementation of the SPP has been disrupted by the COVID-19 pandemic. Further, as a public service the GCMHSS CYMHS experiences frequent staff turn-over and achieving and maintaining fidelity to a model of care can be challenging.

It is also important to acknowledge the limitations of this study. The data represent only a cross-sectional snapshot of the first years of SPP use at one CYMHS. There were limited opportunities to compare practice and outcomes prior to, and after, SPP implementation. The study reports on routinely collected clinical data, much of which was manually extracted, rather than research data. We considered suicide attempt as a key outcome measure, and did not consider suicide re-attempts. We also chose not to include follow-up data, as these presentations were fairly reiterative of initial care provision; incorporating the same components, reflecting on previous strategies, and filling the gaps.

The study utilized data from 2020 as a representative model. This may have introduced bias due to COVID-19 and other potential factors present in a single year rather than choosing to average the findings across years. A future study looking at post COVID results may be beneficial to explore this model further.

Finally, we made no distinction between children who presented with acute episodes of suicidality versus children who presented with chronic suicidality. The latter cohort are placed on the SPP as a first step, but no additional interventions specifically to mitigate chronicity are currently offered. Research in adults shows that interventions such as Acceptance and Commitment Therapy (42) and Cognitive Behavioral Therapy (CBT) (43) may be effective for suicidality. Preliminary evidence in children and adolescents suggests that dialectical behavioral therapies may be beneficial for reducing self-harm and suicidal ideation in young people (44). The use of these more in-depth interventions alongside the brief interventions offered in the SPP to manage suicidality, particularly in children who present with chronic suicidality, is an important area for future research.

## 5 Conclusions

Suicide in children is a significant and unacceptable problem worldwide. This paper provides a descriptive overview of the children presenting in the first five years (December 2016-2021) of the implementation of the Zero Suicide Framework and Suicide Prevention Pathway at a Child and Youth Mental Health Service in Queensland, Australia. It shows that in a service with numerous and diverse presentations, a standardized approach to suicide prevention improves consistency in the delivery of first line interventions. This is a crucial component for improving support for children at risk of suicide.

## Data Availability

The raw data supporting the conclusions of this article will be made available by the authors upon request.

## References

[1] Centers for Disease Control and Prevention. Suicide prevention(2022). Available online at: https://www.cdc.gov/suicide/facts/index.html (Accessed June 29, 2022).

[2] World Health Organisation. Suicide worldwide in 2019: global health estimates. Geneva: World Health Organization (2021).

[3] Australian Institute of Health and Welfare. Deaths by suicide among young people. (2022). Available online at: https://www.aihw.gov.au/suicide-self-harm-monitoring/data/populations-age-groups/suicide-among-young-people (Accessed December 10, 2022).

[4] World Health Organization. Suicide(2021). Available online at: https://www.who.int/news-room/fact-sheets/detail/suicide (Accessed June 29, 2022).

[5] Australian Institute of Health and Welfare. Suicide & self-harm monitoring: Intentional self-harm hospitalisations among young people. Canberra: AIHW (2024). Available at: https://www.aihw.gov.au/suicide-self-harm-monitoring/data/populations-age-groups/intentional-self-harm-hospitalisations-among-young.

[6] Lawrence D Johnson S Hafekost J Boterhoven De Haan K Sawyer M Ainley J. The Mental Health of Children and Adolescents: Report on the second Australian Child and Adolescent Survey of Mental Health and Wellbeing. Canberra: Department of Health (2015).

[7] Stapelberg N Sveticic J Hughes I Turner K. Suicidal presentations to emergency departments in a large Australian public health service over 10 years. Int J Environ Res Public Health. (2020) 17:1–12. doi: 10.3390/ijerph17165920 PMC7460475 32824052

[8] Queensland Government - Gold Coast Health. Child and youth mental health service(2022). Available online at: https://www.goldcoast.health.qld.gov.au/our-services/childrens-services/child-and-youth-mental-health-cymhs (Accessed June 29, 2022).

[9] Stapelberg N Sveticic J Hughes I Almeida-Crasto A Gaee-Atefi T Gill N. Efficacy of the Zero Suicide framework in reducing recurrent suicide attempts: Cross-sectional and time-to-recurrent-event analyses. Br J Psychiatry. (2021) 219:427–36. doi: 10.1192/bjp.2020.190 33176895

[10] Covington D Hogan M Abreu J Berman A Breux P Coffey E. Suicide care in systems framework(2011). Available online at: https://www.sprc.org/resources-programs/suicide-care-systems-frameworks (Accessed June 29, 2022).

[11] Zero Suicide. Study: Zero Suicide practices reduce suicides(2022). Available online at: https://zerosuicide.edc.org/ (Accessed June 29, 2022).

[12] Mokkenstorm J Kerkhof A Smit J Beekman A. Is it rational to pursue zero suicides among patients in health care? Suicide Life-Threatening Behav. (2017) 48:745–54. doi: 10.1111/sltb.12396 PMC6586166 29073324

[13] Turner K Sveticic J Almeida-Crasto A Gaee-Atefi T Green V Grice D. Implementing a systems approach to suicide prevention in a mental health service using the Zero Suicide Framework. Aust New Z J Psychiatry. (2020) 55:241–253. doi: 10.1177/0004867420971698 33198477

[14] Pisani AR Murrie DC Silverman MM. Reformulating suicide risk formulation: from prediction to prevention. Acad Psychiatry. (2016) 40:623–9. doi: 10.1007/s40596-015-0434-6 PMC4937078 26667005

[15] Branjerdporn G McCosker L Jackson D McDowell S Williams P Chand S. The implementation of a zero-suicide framework in a child and youth mental health service in Australia: Processes and learnings. Front Psychiatry. (2024) 15. doi: 10.3389/fpsyt.2024.1370256 PMC11138260 38818025

[16] Ross V Mathieu S Hagwood J Turner K Stapelberg N Welch M. Consumer and carer perspectives of a zero suicide prevention program: A qualitative study. Int J Environ Res Public Health. (2021) 18:10634. doi: 10.3390/ijerph182010634s 34682380 PMC8535550

[17] Pagura J Cox BJ Sareen J Enns MW. Factors associated with multiple versus single episode suicide attempts in the 1990-1992 and 2001-2003 United States national comorbidity surveys. J Nerv Ment Dis. (2008) 196:806–13. doi: 10.1097/NMD.0b013e31818b6a77 19008731

[18] Yuan J. HoNOSCA in an adolescent psychiatric inpatient unit: an exploration of outcome measures(2015). Available online at: https://www.psychiatria-danubina.com/UserDocsImages/pdf/dnb_vol27_sup1/dnb_vol27_sup1_357.pdf (Accessed July 01, 2022). 26417796

[19] Volkmar F. Encyclopedia of Autism Spectrum Disorders. New York: Springer (2020).

[20] Hall C Guo B Valentine A Groom M Daley D Sayal K. The validity of the Strengths and Difficulties Questionnaire (SDQ) for children with ADHD symptoms. PloS One. (2019) 14:e0218518. doi: 10.1371/journal.pone.0218518 31216327 PMC6583960

[21] Australian Bureau of Statistics [ABS]. Socio-Economic Indexes for Areas. Canberra: ABS (2016). Available at: https://www.abs.gov.au/websitedbs/censushome.nsf/home/seifa.

[22] IBM. IBM SPSS Statistics 28. New York, USA: IBM (2021). Available at: https://www.ibm.com/docs/en/spss-statistics/28.0.0?topic=overview-whats-new-in-version-28.

[23] Australian Institute of Health and Welfare. Suicide and self-harm monitoring: The use of mental health services, psychological distress, loneliness, suicide, ambulance attendances and COVID-19(2022). Available online at: https://www.aihw.gov.au/suicide-self-harm-monitoring/data/covid-19 (Accessed June 29, 2022).

[24] Sveticic J Stapelberg N Turner K. Suicide prevention during COVID-19: Identification of groups with reduced presentations to emergency departments. Australas Psychiatry. (2021) 29:333–336. doi: 10.1177/1039856221992632 33626306

[25] Australian Institute of Health and Welfare. Suicide and self-harm monitoring: Deaths by suicide over time(2022). Available online at: https://www.aihw.gov.au/suicide-self-harm-monitoring/data/deaths-by-suicide-in-Australia/suicide-deaths-over-time (Accessed June 29, 2022).

[26] Australian Institute of Health and Welfare. Suicide and intentional self-harm(2020). Available online at: https://www.aihw.gov.au/reports/Australias-health/suicide-and-intentional-self-harms (Accessed June 29, 2022).

[27] Lee S Dwyer J Paul E Clarke D Treleaven S Roseby R. Differences by age and sex in adolescent suicide. Aust New Z J Public Health. (2019) 43:248–53. doi: 10.1111/1753-6405.12877 30786107

[28] Australian Institute of Health and Welfare. Suicide and self-harm monitoring: Social and economic factors and deaths by suicide(2022). Available online at: https://www.aihw.gov.au/suicide-self-harm-monitoring/data/behaviours-risk-factors/social-factors-suicide (Accessed June 29, 2022).

[29] Australian Bureau of Statistics. Gold Coast: 2021 Census Aboriginal and/or Torres Strait Islander people QuickStats. (2022). Available online at: https://www.abs.gov.au/census/find-census-data/quickstats/2021/AUS (Accessed November 5, 2022).

[30] City of Gold Coast. Statistics for Aboriginal and Torres Strait Islander peoples(2022). Available online at: https://www.goldcoast.qld.gov.au/Council-region/About-our-city/Population-data/Statistics-for-Aboriginal-Torres-Strait-Islander-Peoples (Accessed June 29, 2022).

[31] Australian Institute of Health and Welfare. Suicide and self-harm monitoring: Intentional self-harm hospitalisations among First Nations people. Canberra: AIHW (2023). Available at: https://www.aihw.gov.au/suicide-self-harm-monitoring/data/populations-age-groups/intentional-self-harm-hospitalisations-indigenous.

[32] Australian Institute of Health and Welfare. Mental health(2020). Available online at: https://www.aihw.gov.au/reports/Australias-health/mental-health (Accessed June 29, 2022).

[33] Carter G Milner A McGill K Pirkis J Kapur N Spittal M. Predicting suicidal behaviours using clinical instruments: Systematic review and meta-analysis of positive predictive values for risk scales. Br J Psychiatry. (2017) 210:387–95. doi: 10.1192/bjp.bp.116.182717 28302700

[34] Cwik M O’Keefe V Haroz E. Suicide in the pediatric population: Screening, risk assessment and treatment. Int Rev Psychiatry. (2020) 32:254–26. doi: 10.1080/09540261.2019.1693351 PMC7190447 31922455

[35] Turner K Stapelberg N Sveticic J Pisani A. Suicide risk classifications do not identify those at risk: Where to from here? Australas Psychiatry. (2022) 30:139. doi: 10.1177/10398562211032233 PMC8894938 34339610

[36] Liang J Kõlves K Lew B De Leo D Yuan L Talib M. Coping strategies and suicidality: A cross-sectional study from China. Front Psychiatry. (2020) 11. doi: 10.3389/fpsyt.2020.00129 PMC7083072 32231596

[37] Balazs J Kereszteny A. Attention-deficit/hyperactivity disorder and suicide: A systematic review. World J Psychiatry. (2017) 7:44–59. doi: 10.5498/wjp.v7.i1.44 28401048 PMC5371172

[38] Klomek A Souranger A Niemela S Kumpulainen K Piha J Tamminen T. Childhood bullying behaviors as a risk for suicide attempts and completed suicides: a population-based birth cohort study. J Am Acad Child Adolesc Psychiatry. (2009) 48:254–61. doi: 10.1097/CHI.0b013e318196b91f 19169159

[39] Bao W Qian Y Fei W Tian S Geng Y Wang S. Bullying victimization and suicide attempts among adolescents in 41 low- and middle-income countries: Roles of sleep deprivation and body mass. Front Public Health. (2023) 11. doi: 10.3389/fpubh.2023.1064731 PMC9992427 36908401

[40] Levy T Kil H Schachar R Itzhaky L Andrade B. Suicidality risk in children and adolescents with externalizing disorders: symptoms profiles at high risk and the moderating role of dysregulated family relationships. Eur Child Adolesc Psychiatry. (2023) 33:811–820. doi: 10.1007/s00787-023-02190-z 37043094

[41] Soole R Kõlves K De Leo D. Suicide in children: A systematic review. Arch Suicide Res. (2015) 19:285–304. doi: 10.1080/13811118.2014.996694 25517290

[42] Ducasse D Jaussent I Arpon-Brand V Vienot M Laglaoui C Beziat S. Acceptance and commitment therapy for the management of suicidal patients: A randomised controlled trial. Psychother Psychosomatics. (2018) 87:211–22. doi: 10.1159/000488715 29874680

[43] Mewton L Andrews G. Cognitive behavioral therapy for suicidal behaviors: improving patient outcomes. Psychol Res Behav Manage. (2016) 9:21–9. doi: 10.2147/PRBM.S84589 PMC4780394 27042148

[44] Bahji A Pierce M Wong J Roberge JN Ortega I Patten S. Comparative efficacy and acceptability of psychotherapies for self-harm and suicidal behavior among children and adolescents: A systematic review and network meta-analysis. JAMA Netw Open. (2021) 4:e216614. doi: 10.1001/jamanetworkopen.2021.6614 33861328 PMC8052594

